# Nod2-Nodosome in a Cell-Free System: Implications in Pathogenesis and Drug Discovery for Blau Syndrome and Early-Onset Sarcoidosis

**DOI:** 10.1155/2016/2597376

**Published:** 2016-06-15

**Authors:** Tomoyuki Iwasaki, Naoe Kaneko, Yuki Ito, Hiroyuki Takeda, Tatsuya Sawasaki, Toshio Heike, Kiyoshi Migita, Kazunaga Agematsu, Atsushi Kawakami, Shinnosuke Morikawa, Sho Mokuda, Mie Kurata, Junya Masumoto

**Affiliations:** ^1^Department of Pathology, Ehime University Proteo-Science Center and Graduate School of Medicine, Shitsukawa 454, Toon, Ehime 791-0295, Japan; ^2^Division of Cell-Free Sciences, Ehime University Proteo-Science Center, Bunkyocho 3, Matsuyama, Ehime 790-8577, Japan; ^3^Department of Pediatrics, Kyoto University Graduate School of Medicine, Shogoin Kawaramachi 54, Kyoto 606-8507, Japan; ^4^Clinical Research Center, Nagasaki Medical Center, Kubara 2-1001-1, Omura, Nagasaki 856-8562, Japan; ^5^Department of Infection and Host Defense, Shinshu University Graduate School of Medicine, Asahi 3-1-1, Matsumoto, Nagano 390-8621, Japan; ^6^Unit of Translational Medicine, Department of Immunology and Rheumatology, Nagasaki University Graduate School of Biomedical Sciences, Medicine, Sakamoto 1-7-1, Nagasaki 852-8501, Japan

## Abstract

Nucleotide-binding oligomerization domain-containing protein (Nod) 2 is an intracellular pattern recognition receptor, which recognizes muramyl dipeptide (N-Acetylmuramyl-L-Alanyl-D-Isoglutamine: MDP), a bacterial peptidoglycan component, and makes a NF-*κ*B-activating complex called nodosome with adaptor protein RICK (RIP2/RIPK2). Nod2 mutants are associated with the autoinflammatory diseases, Blau syndrome (BS)/early-onset sarcoidosis (EOS). For drug discovery of BS/EOS, we tried to develop Nod2-nodosome in a cell-free system. FLAG-tagged RICK, biotinylated-Nod2, and BS/EOS-associated Nod2 mutants were synthesized, and proximity signals between FLAG-tagged and biotinylated proteins were detected by amplified luminescent proximity homogeneous assay (ALPHA). Upon incubation with MDP, the ALPHA signal of interaction between Nod2-WT and RICK was increased in a dose-dependent manner. The ALPHA signal of interaction between RICK and the BS/EOS-associated Nod2 mutants was more significantly increased than Nod2-WT. Notably, the ALPHA signal between Nod2-WT and RICK was increased upon incubation with MDP, but not when incubated with the same concentrations, L-alanine, D-isoglutamic acid, or the MDP-D-isoform. Thus, we successfully developed Nod2-nodosome in a cell-free system reflecting its function in vivo, and it can be useful for screening Nod2-nodosome-targeted therapeutic molecules for BS/EOS and granulomatous inflammatory diseases.

## 1. Introduction

Nucleotide-binding oligomerization domain-containing protein (Nod) 2 is a nuclear factor- (NF-) *κ*B-activating intracellular pattern recognition receptor, which was identified as a susceptibility gene product of Crohn's disease, an inflammatory bowel disease [1–3].

Nod2 was reported to be oligomerized with adaptor protein RICK (RIP2/RIPK2) and IKK complexes, which can activate NF-*κ*B by muramyl dipeptide (N-Acetylmuramyl-L-Alanyl-D-Isoglutamine: MDP), one of the components of bacterial cell-wall peptidoglycan, and is utilized as an immune-stimulatory adjuvant for vaccination and for developing antibodies [4–8].

It was also discovered that an autoinflammatory disease, Blau syndrome (BS)/early-onset sarcoidosis (EOS), was caused by a point mutation of Nod2, encoding a constitutively active form, resulting in NF-*κ*B activation [9–11]. BS/EOS is a systemic granulomatous disease, and patients with BS/EOS have nonnecrotizing granulomas of the skin, eyes, and joints [12]. Other granulomatous inflammatory diseases were similarly reported to be associated with Nod2 [13, 14]. Therefore, Nod2 has been thought to be involved in granulomatous disorders [15] and may be an attractive drug target for treatment of these chronic inflammatory granulomatous diseases.

Thus, we aimed to develop a reconstituted protein-protein interaction assay system between wild-type Nod2 and the BS/EOS-associated mutants of Nod2 and RICK in a cell-free system, further called the reconstituted Nod2-nodosome in a cell-free system [16].

## 2. Materials and Methods

### 2.1. Plasmid Construction, Site-Specific Mutagenesis for the BS/EOS-Associated Mutant Nod2, and Protein Synthesis

The cDNA of human Nod2 and RICK were derived from pcDNA3-Nod2 and pcDNA3-RICK, kindly provided by Dr. G Núñez (accession number: AF178930 and AF027706). Each open reading frame, without stop codons, was modified in a two-step polymerase chain reaction (PCR). Briefly, the initial PCR product for Nod2-WT was amplified using the primers: forward S1-Nod2_F: 5′-CCACCCACCACCACCAATGGGGGAAGAGGGTGGTTCAG-3′ and reverse Nod2-T1(F)_R: 5′-TCCAGCACTAGCTCCAGAAAGCAAGAGTCTGGTGTCCCT-3′. The initial PCR product for Nod2-CARD1+CARD2 (CARDs) was amplified using the primers: forward S1-Nod2_F: 5′-CCACCCACCACCACCAATGGGGGAAGAGGGTGGTTCAG-3′ and reverse Nod2-T1(CARDs)_R: 5′-TCCAGCACTAGCTCCAGACTTGCATGTGGCAGCTTCCA-3′.

The initial PCR product for RICK-WT was amplified using the primers: forward S1-RICK_F: 5′-CCACCCACCACCACCAATGAACGGGGAGGCCATCTG-3′ and reverse RICK-T1(F)_R: 5′-TCCAGCACTAGCTCCAGACATGCTTTTATTTTGAAGTAAATTTA -3′. The initial PCR product for RICK-CARD was amplified using the primers: forward S1-RICK (CARD)_F: 5′-CCACCCACCACCACCAATGAACGGGGAGGCCATCTG-3′ and reverse RICK-T1(F)_R: 5′-TCCAGCACTAGCTCCAGACATGCTTTTATTTTGAAGTAAATTTA-3′.

The second PCR was carried out using the following primer set: attB1-S1: 5′-GGGGACAAGTTTGTACAAAAAAGCAGGCTTCCACCCACCACCACCAATG-3′ and attB2-T1: 5′-GGGGACCACTTTGTACAAGAAAGCTGGGTCTCCAGCACTAGCTCCAGA-3′ with the initial PCR products as templates. These primers are shown in Table 1.

**Figure 1:**
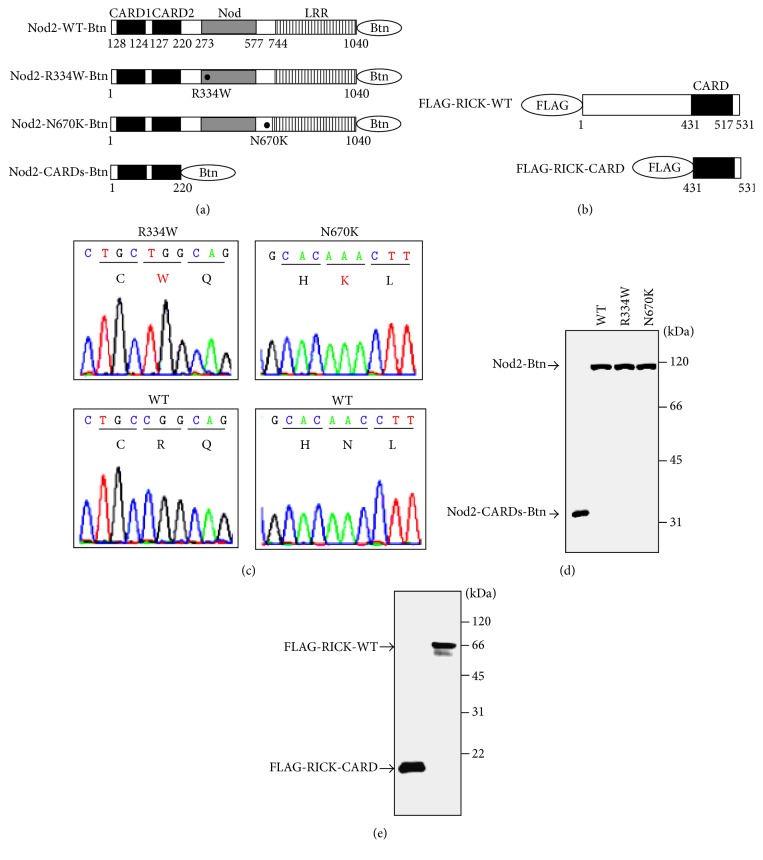
Schematic representation of Nod2 and RICK, and sequencing chromatograms of Nod2 plasmids containing the BS/EOS-associated mutations, and protein syntheses. (a) Schematic representations of biotinylated wild-type and mutant Nod2. C-terminal biotinylated full-length wild-type Nod2 (Nod2-WT-Btn), full-length R334W-mutated Nod2 (Nod2-R334W-Btn), full-length N670K-mutated Nod2 (Nod2-N670K-Btn), and tandem CARD1- and CARD2-domains-only Nod2 (Nod2-CARDs) are indicated. (b) Schematic representations of FLAG-tagged wild-type and CARD-domain-only RICK. N-terminal FLAG-tagged full-length RICK (FLAG-RICK-FL) and CARD-domain-only RICK (FLAG-RICK-CARD) are indicated. The caspase recruitment domain (CARD) is indicated by black boxes. The nucleotide-binding oligomerization domain-containing protein (Nod) is indicated by grey boxes. Leucine-rich repeats are indicated by striped boxes. Amino acid sequence number and mutated amino acids are indicated under each schema. (c) Sequencing chromatograms of Nod2 and mutated-Nod2 plasmids. The wild-type and mutated-Nod2 plasmids pDONR221-Nod2-WT, pDONR221-Nod2-R334W, and pDONR221-Nod2-N670K were sequenced to confirm (from CGG to TGG corresponding to R334W in the right panel; from AAC to AAA corresponding to N670K in the left panel) mutations at the appropriate site. (d) Western blotting analysis of biotinylated Nod2 and its mutants. A total of 1.5 *μ*g of synthetic protein was subjected to SDS-PAGE followed by Western blotting. Protein detection on the membranes was performed using HRP-conjugated streptavidin. Molecular weights are indicated at right. (e) Western blotting analysis of FLAG-tagged RICK and CARD-domain-only RICK. A total of 1.5 *μ*g of synthetic protein was subjected to SDS-PAGE followed by Western blotting. Protein detection on the membranes was performed using anti-FLAG mAb M2. Molecular weights are indicated at right.

PCR-based site-specific mutagenesis for Nod2-R334W was amplified by two-step PCR using the following primer sets: forward S1-Nod2_F: 5′-CCACCCACCACCACCAATGGGGGAAGAGGGTGGTTCAG-3′ and reverse Nod2-R334W_R: 5′-GCACTGCAGCTGCCAGCAGCTGAATGGGAA-3′, and forward Nod2-R334W_F: 5′-TTCCCATTCAGCTGCTGGCAGCTGCAGTGC-3′ and reverse Nod2-T1(F)_R: 5′-TCCAGCACTAGCTCCAGAAAGCAAGAGTCTGGTGTCCCT-3′ for the first-step overlapping DNA fragment set. PCR-based site-specific mutagenesis for Nod2-N670K was also amplified by two-step PCR using the following primer sets: forward S1-Nod2_F: 5′-CCACCCACCACCACCAATGGGGGAAGAGGGTGGTTCAG-3′ and reverse Nod2-N670K_R: 5′-TGCTGTGATCTGAAGTTTGTGCGGCTCGGC-3′, and forward Nod2-N670K_F: 5′-GCCGAGCCGCACAAACTTCAGATCACAGCA-3′ and reverse Nod2-T1(F)_R: 5′-TCCAGCACTAGCTCCAGAAAGCAAGAGTCTGGTGTCCCT-3′ for the first-step overlapping DNA fragment set. R334W and N670K mutated-Nod2 DNA fragments were amplified by a second PCR step using the following primer set: attB1-S1: 5′-GGGGACAAGTTTGTACAAAAAAGCAGGCTTCCACCCACCACCACCAATG-3′ and attB2-T1: 5′-GGGGACCACTTTGTACAAGAAAGCTGGGTCTCCAGCACTAGCTCCAGA-3′, from the first-step overlapping DNA fragment sets as template. These oligonucleotide sequences for mutated Nod2 are listed in Table 1. PCR products were inserted into a pDONR221 vector using Gateway® BP Clonase® II Enzyme mix (Life Technologies, Carlsbad, CA, USA) to generate entry clones. Nod2 entry clones, pDONR221-Nod2-WT, pDONR221-Nod2-R334W, pDONR221-Nod2-N670K, and pDONR221-Nod2-CARDs, were inserted into pEU-E01-GW-bls-STOP for cell-free protein expression. RICK entry clones, pDONR221-RICK-WT and pDONR221-RICK-CARD, were inserted into pEU-E01-FLAG-GW-STOP, using the Gateway LR Clonase® II Enzyme mix. These constructs were confirmed by sequencing (Figure 1(c)). The constructed plasmids were used to synthesize their respective proteins using the WEPRO1240 Expression Kit (Cell-free, Inc., Matsuyama, Japan) and were followed by Western blotting.

### 2.2. Western Blotting Analysis

A total of 1.5 *μ*g of synthetic protein was subjected to SDS-PAGE followed by Western blotting analysis. Protein detection on the blotting membranes was performed using anti-FLAG mAb M2 (Sigma-Aldrich, St. Louis, MO, USA), followed by peroxidase-conjugated affinity-purified F(ab′)_2_ fragment of goat anti-mouse IgG, F(ab′)_2_ fragment-specific (Jackson ImmunoResearch, West Grove, PA, USA) for FLAG-tagged proteins, or HRP-conjugated streptavidin (Nacalai, Kyoto, Japan) for biotinylated proteins.

### 2.3. Pull-Down Assay

One-microgram biotinylated-Nod2-WT (Nod2-WT-Btn) and 1 *μ*g FLAG-tagged RICK-WT (FLAG-RICK-WT) lysed in 300 *μ*L NP-40 buffer [1% Nonidet P-40, 142.5 mmol/L KCl, 5 mmol/L MgCl_2_, 10 mmol/L HEPES (pH 7.6), 0.2 mmol/L phenylmethylsulfonylfluoride (PMSF), and 1 mmol/L EDTA] were precipitated with 20 *μ*L streptavidin-conjugated agarose beads (Invitrogen) with or without 5.33 mg/mL MDP (Sigma-Aldrich) and incubated for 3 hours at 4°C. The precipitations were subjected to SDS-PAGE and immunoblotting. Detection on the blotting membranes was performed using anti-FLAG mAb M2 (Sigma-Aldrich) or anti-Nod2 mAb 2D9 (Cayman Chemical, Ann Arbor, MI, USA).

### 2.4. Amplified Luminescent Proximity Homogeneous Assay

Synthesized protein-protein interactions were assessed by amplified luminescent proximity homogeneous assay (ALPHA) (PerkinElmer). A total of 100 ng of each protein was added to ALPHA buffer [100 mM Tris-HCl (pH 8.0), 0.01% (v/v) Tween 20], 1 mg/mL BSA, 16.67 *μ*g/mL streptavidin-coated donor beads (PerkinElmer, Waltham, MA, USA), 16.67 *μ*g/mL protein-A-conjugated acceptor beads, and 5 *μ*g/mL anti-FLAG mAb M2 and incubated in a Shallow Well ALPHAPlate-384 (PerkinElmer) at 25°C for 24 hours. Fluorescence emission signals of each well were measured using an EnSpire Multimode Plate Reader (PerkinElmer).

### 2.5. Statistics

Results are presented as the mean and standard deviation. Levels of significance were evaluated using Student's *t*-test. A *p* value < 0.05 was considered statistically significant.

## 3. Results

### 3.1. Recombinant Nod2, RICK, and Their Mutant Proteins Were Successfully Synthesized Using a Wheat Germ Cell-Free System

The schematics of the C-terminal biotinylated full-length wild-type Nod2 (Nod2-WT-Btn), full-length R334W-mutated Nod2 (Nod2-R334W-Btn), full-length N670K-mutated Nod2 (Nod2-N670K-Btn), and tandem CARD1- and CARD2-domains-only Nod2 (Nod2-CARDs) are shown in Figure 1(a). The schematics of the N-terminal FLAG-tagged full-length RICK (FLAG-RICK-FL) and CARD-domain-only RICK (FLAG-RICK-CARD) are shown in Figure 1(b). The plasmids vectors pDONR221-Nod2-WT, pDONR221-Nod2-R334W, pDONR221-Nod2-N670K, pDONR221-Nod2-CARDs, pDONR221-RICK-WT, and pDONR221-RICK-CARD were sequenced and confirmed. The proper BS/EOS-associated mutation sequences of Nod2 were confirmed (Figure 1(c)). Nod2 and its variants, including Nod2-WT-Btn, Nod2-R334W-Btn, Nod2-N670K-Btn, and Nod2-CARDs, were successfully synthesized using the wheat germ cell-free system (Figure 1(d)). FLAG-RICK-WT and FLAG-RICK-CARD were also successfully synthesized using the wheat germ cell-free system (Figure 1(e)).

### 3.2. MDP-Induced Interaction between Nod2 and RICK in Nodosome in a Cell-Free System

**Figure 2:**
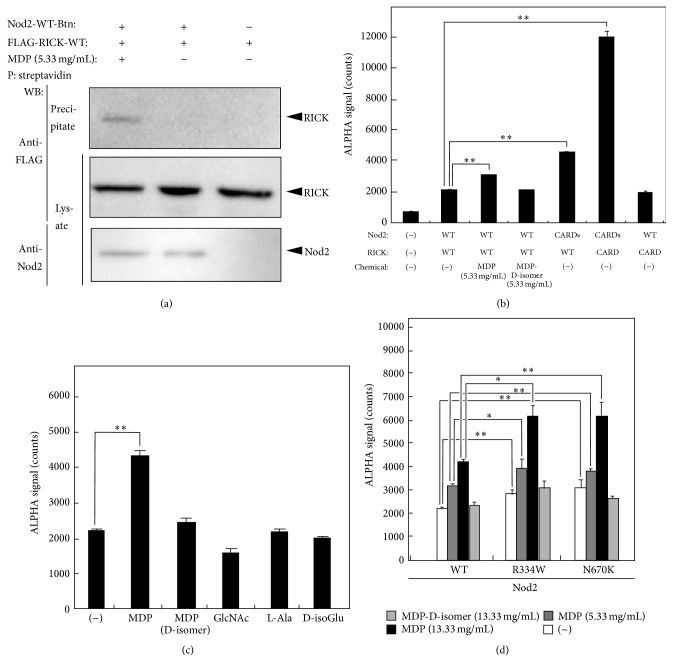
Construction of Nod2-nodosome containing the BS/EOS-associated mutation in a cell-free system. Synthetic protein-protein interactions were detected by pull-down assay and amplified luminescent proximity homogeneous assay (ALPHA). (a) Biotinylated-Nod2-WT (Nod2-WT-Btn) and 1 *μ*g FLAG-tagged RICK-WT (FLAG-RICK-WT) lysed in 300 *μ*L NP-40 buffer were precipitated with 20 *μ*L streptavidin-conjugated agarose beads with or without MDP. The precipitations were subjected to SDS-PAGE and immunoblotting. Detection on the blotting membranes was performed using anti-FLAG mAb M2 or anti-Nod2 mAb. (b) A total of 100 ng of each protein indicated was incubated with 5 *μ*g/mL anti-FLAG mAb M2, 16.67 *μ*g/mL protein-A-conjugated ALPHA acceptor beads, and 16.67 *μ*g/mL streptavidin-conjugated ALPHA donor beads for 24 hours, with or without 5.33 mg/mL MDP or 5 mg/mL N-Acetylmuramyl-D-Alanyl-D-Isoglutamine (MDP-D-isomer). Responses (counts) were measured using the EnSpire*™* Multimode Plate Reader. (c) Activation of Nod2-nodosome in a cell-free system by MDP degradation components. Interactions between Nod2-WT-Btn and FLAG-RICK-WT were detected by ALPHA. A total of 100 ng of Nod2-WT-Btn and FLAG-RICK-WT were incubated with 5 *μ*g/mL anti-FLAG mAb M2, 16.67 *μ*g/mL protein-A-conjugated ALPHA acceptor beads and 16.67 *μ*g/mL streptavidin-conjugated ALPHA donor beads for 24 hours, without or with 5.33 mg/mL MDP, 5.33 mg/mL MDP (D-isomer), 5.33 mg/mL N-acetylglucosamine (GlcNAc), 5.33 mg/mL L-alanine (L-Ala), or 5.33 mg/mL D-isoglutamine (D-isoGlu). Responses (counts) were measured using the EnSpire Multimode Plate Reader. The results are representative of three independent experiments and given as means ± standard deviation from triplicate wells. (d) A total of 100 ng of Nod2-WT-Btn, or Nod2-R334W-Btn, or Nod2-N670K-Btn with FLAG-RICK-WT was incubated with 5 *μ*g/mL anti-FLAG mAb M2, 16.67 *μ*g/mL protein-A-conjugated ALPHA acceptor beads, and 16.67 *μ*g/mL streptavidin-conjugated ALPHA donor beads for 24 hours, with or without 5.33 mg/mL or 13.33 mg/mL MDP or 13.33 mg/mL MDP-D-isomer. The results are representative of three independent experiments and given as means ± standard deviation from triplicate wells. CARD, caspase recruitment domain; MDP, muramyl dipeptide; WT, wild-type; P, precipitation; WB, western blot. ^*∗*^ *p* value < 0.05 and ^*∗∗*^ *p* value < 0.01 were considered statistically significant using Student's *t*-test.

Nod2-WT-Btn was coprecipitated with FLAG-RICK-WT when incubated with 5.33 mg/mL MDP, but not without MDP in pull-down assay (Figure 2(a)). The baseline ALPHA signal of Nod2-WT-Btn and FLAG-RICK-WT interaction was approximately 2000 counts, with no stimulation (Figure 2(b)). The ALPHA signal of interaction between Nod2-WT-Btn and FLAG-RICK-WT increased upon incubation with 5.33 mg/mL MDP (Sigma-Aldrich, St. Louis, MO, USA) (*p* < 0.01), whereas no increase was detected upon incubation with the same amount (5.33 mg/mL) of N-Acetylmuramyl-D-Alanyl-D-Isoglutamine (MDP-D-isomer) (Invivogen, San Diego, CA, USA) (Figure 2(b)). The ALPHA signal of interaction between Nod2-CARDs-Btn and FLAG-RICK-WT increased without MDP (*p* < 0.01). Furthermore, the ALPHA signal of interaction between Nod2-CARDs-Btn and FLAG-RICK-CARD without MDP was significantly increased (*p* < 0.01), whereas the ALPHA signal of interaction between Nod2-WT-Btn and FLAG-RICK-CARD was not increased and was similar to baseline levels (Figure 2(b)).

### 3.3. The MDP Degradation Products Did Not Induce Interaction between Nod2 and RICK in the Cell-Free System

Using the Nod2-nodosome in a cell-free system established above, we assessed whether MDP degradation products, such as L-alanine, D-isoglutamic acid, and N-acetylglucosamine (GlcNAc), could induce interaction between Nod2 and RICK in the cell-free system. No increased signals were observed upon incubation with a negative control (−), MDP-D-isomer, L-alanine, D-isoglutamic acid, and GlcNAc (Figure 2(c)).

### 3.4. ALPHA Signal in Response to MDP Was Observed in Nod2-Nodosome in a Cell-Free System Containing the BS/EOS-Associated Mutations

To test the activity of Nod2-nodosome in a cell-free system with or without BS/EOS-associated mutations, we assessed the interaction between FLAG-RICK-WT and Nod2-WT-Btn, Nod2-R334W-Btn, or Nod2-N670K-Btn, in a cell-free system. The ALPHA signal between FLAG-RICK-WT and Nod2-WT-Btn increased upon incubation with 5.33 mg/mL and 13.33 mg/mL MDP, in a dose-dependent manner (Figure 2(d)). The baseline ALPHA signal between the BS/EOS-associated Nod2 mutants, such as Nod2-R334W-Btn and Nod2-N670K-Btn, and FLAG-RICK-WT with no stimulation was higher than that between Nod2-WT-Btn and FLAG-RICK-WT (*p* < 0.01, *p* < 0.01, resp.) (Figure 2(d), left columns). Upon incubation with 5.33 mg/mL, the ALPHA signal between Nod2-WT-Btn and FLAG-RICK-WT increased in a dose-dependent manner, and signals between Nod2-R334W-Btn or Nod2-N670K-Btn and FLAG-RICK-WT were more significantly increased (*p* < 0.05, *p* < 0.01, resp.) (Figure 2(d), center columns). Upon incubation with 13.33 mg/mL MDP, the ALPHA signal between Nod2-WT-Btn and FLAG-RICK-WT increased in a dose-dependent manner, and signals between Nod2-R334W-Btn or Nod2-N670K-Btn and FLAG-RICK-WT were more significantly increased (*p* < 0.05, *p* < 0.01, resp.) (Figure 2(d), right columns).

## 4. Discussion

Autoinflammatory syndromes are known to stem from aberrant innate immune complex disorders, some of which are known to be due to the excessive innate immune activation by mutations of the pathogen-recognizing receptors [17]. BS/EOS is one of the autoinflammatory syndromes, which is characterized by systemic chronic granulomatous inflammation, resulting in nonnecrotizing granuloma of the skin, eyes, and joints [12]. The susceptibility gene product of BS/EOS has been identified to be Nod2/CARD15 [11, 18].

In the present study, we developed Nod2-nodosomes containing wild-type and the BS/EOS-associated mutants R334W and N670K in a cell-free system, assessed by ALPHA. In general, recombinant protein synthesis has been used for bacterial or viral systems, such as* Escherichia coli*, for structural and functional studies. However, synthetic proteins sometimes are unable to be investigated, because many proteins are expressed in an insoluble form [19]. Therefore, we employed the wheat germ cell-free protein synthesis system, and Nod2-WT-Btn, Nod2-R334W-Btn, Nod2-N670K-Btn, Nod2-CARDs, FLAG-RICK-WT, and FLAG-RICK-CARD were successfully synthesized (Figure 1). To date, we reported the generation of AIM2-inflammasome in a cell-free system, which is another intracellular pattern recognition receptor with adaptor ASC oligomerization, which was suitable for wheat germ cell-free protein synthesis [20]. These results suggested that the wheat germ cell-free protein synthesis system has an advantage for reconstituting intracellular pattern recognition receptor complexes for resolving innate immune complex-mediated autoinflammatory diseases.

First, we confirmed whether the Nod2-nodosome could recognize the bacterial cell-wall component MDP, which is a previously reported Nod2-ligand [6, 7]. As shown in Figure 2(a), Nod2-WT-Btn was coprecipitated with FLAG-RICK-WT when incubated with MDP, but not without MDP in pull-down assay (Figure 2(a)). That was consistent with ALPHA in Figure 2(b). As shown in Figure 2(b), the ALPHA signal of interaction between Nod2-WT-Btn and FLAG-RICK-WT was increased upon incubation with MDP, but not without a ligand or with the MDP-D-isomer or MDP-degraded components (Figures 2(b) and 2(c)). These results indicated that Nod2-nodosome in a cell-free system is capable of detecting for its specific ligand, MDP.

Next, we tested whether the BS/EOS-associated Nod2 mutations induced increased activity. As shown in Figure 2(d), the ALPHA signal of interaction between FLAG-RICK-WT and Nod2-R334W-Btn or Nod2-N670K-Btn was much higher than when with Nod2-WT-Btn, with or without MDP stimulation (Figure 2(d)). These results are consistent with previous studies on the clinical manifestations of BS/EOS [21], suggesting that Nod2-nodosome in a cell-free system may suitably reflect the characteristics of endogenous Nod2-nodosome.

It is noted that there are some limitations of nodosome in a cell-free system. Only the initial event of Nod2-RICK interaction is detected. Upstream, downstream, and regulatory events in the activation of Nod2 cannot be measured with the nodosome in a cell-free system, as presented.

In conclusion, we developed Nod2-nodosome in a cell-free system, assessed by ALPHA. Nod2-nodosomes containing the BS/EOS-associated mutations Nod2-R334W and Nod2-N670K were more sensitive to MDP than Nod2-WT. Therefore, the Nod2-nodosomes in a cell-free system developed in the present study can be a useful tool for further investigation of pathogenesis of BS/EOS and discovery of its therapeutics.

## Figures and Tables

**Table 1:** Oligonucleotide sequences for plasmid construction.

Primer name	Primer sequence
S1-Nod2_F	5′-CCACCCACCACCA **CCAATGG**GGGAAGAGGGTGGTTCAG-3′
Nod2-T1(F)_R	5′-TCCAGCACTAGCTCCAGAAAGCAAGAGTCTGGTGTCCCT-3′
Nod2-T1(CARDs)_R	5′-TCCAGCACTAGCTCCAGACTTGCATGTGGCAGCTTCCA-3′
S1-RICK_F	5′-CCACCCACCACCA **CCAATGA**ACGGGGAGGCCATCTG-3′
RICK-T1(F)_R	5′-TCCAGCACTAGCTCCAGACATGCTTTTATTTTGAAGTAAATTTA-3′
S1-RICK(CARD)_F	5′-CCACCCACCACCA **CCAATGC**TGCAGCCTGGTATAGCCC-3′
attB1-S1	5′-GGGGACAAGTTTGTACAAAAAAGCAGGCTTCCACCCACCACCACCAATG-3′
attB2-T1	5′-GGGGACCACTTTGTACAAGAAAGCTGGGTCTCCAGCACTAGCTCCAGA-3′
Nod2-R334W_F	5′-TTCCCATTCAGCTGC*TGG*CAGCTGCAGTGC-3′
Nod2-R334W_R	5′-GCACTGCAGCTG*CCA*GCAGCTGAATGGGAA-3′
Nod2-N670K_F	5′-GCCGAGCCGCAC*AAA*CTTCAGATCACAGCA-3′
Nod2-N670K_R	5′-TGCTGTGATCTGAAG*TTT*GTGCGGCTCGGC-3′

Underline indicates S1 or T1 sequence.

Bold indicates Kozak consensus sequence.

Italic indicates mutation codons for specific amino acids.
